# The Implications of ncRNAs in the Development of Human Diseases

**DOI:** 10.3390/ncrna7010017

**Published:** 2021-02-24

**Authors:** Elena López-Jiménez, Eduardo Andrés-León

**Affiliations:** 1 Centre for Haematology, Immunology and Inflammation Department, Faculty of Medicine, Imperial College London, London W12 0NN, UK; 2 Unidad de Bioinformática, Instituto de Parasitología y Biomedicina “López-Neyra”, Consejo Superior de Investigaciones Científicas, 18016 Granada, Spain

**Keywords:** ncRNAs, lncRNAs, circRNAs, piRNAs, miRNAs in cancer, cardiovascular and neurodegenerative diseases

## Abstract

The mammalian genome comprehends a small minority of genes that encode for proteins (barely 2% of the total genome in humans) and an immense majority of genes that are transcribed into RNA but not encoded for proteins (ncRNAs). These non-coding genes are intimately related to the expression regulation of protein-coding genes. The ncRNAs subtypes differ in their size, so there are long non-coding genes (lncRNAs) and other smaller ones, like microRNAs (miRNAs) and piwi-interacting RNAs (piRNAs). Due to their important role in the maintenance of cellular functioning, any deregulation of the expression profiles of these ncRNAs can dissemble in the development of different types of diseases. Among them, we can highlight some of high incidence in the population, such as cancer, neurodegenerative, or cardiovascular disorders. In addition, thanks to the enormous advances in the field of medical genomics, these same ncRNAs are starting to be used as possible drugs, approved by the FDA, as an effective treatment for diseases.

## 1. Introduction

New high-throughput platforms, such as next generation sequencing (NGS), have revolutionized our knowledge of the function and the organization within the eukaryotic genome. In fact, it has provided us totally unexpected information, for example, the mammalian genome transcription rate is much higher than expected, close to 80% [1]. More surprisingly is that most of these genes that are transcribed into RNA molecules does not encode for proteins. As a result, the study of the function of non-coding RNAs (ncRNAs) in the cell is crucial [2,3]. Moreover, some authors advocate that the degree of complexity of an organism should be measured based on the number of non-coding genes, rather than the number of protein-coding genes (that barely constitute 2% of the whole genome size) [4].

To date, different studies confirm that ncRNA can perform many different functions, mainly divided in regulatory or structural tasks. Among the first ones, we include the regulation of gene expression, both at transcriptional (eRNA) and post-transcriptional (microRNA; miRNA) levels, the protection against external nucleic acids (piRNA) [2,3,4], as well as the regulation of RNA processing (ribozymes) and translation (miRNA). Regarding to the structural functions, ribosomal RNA (rRNA) and transfer RNA (tRNA) were the first non-coding RNAs described, due to their high concentration in the cells [5,6,7]. They are involved in essential roles like protein synthesis and post- and pre-transcriptional regulation of gene expression [8,9]. Please see Table 1.

Due to its very important function within the cellular regulatory system, any failure that leads to the malfunction of any of the forementioned non-coding RNAs, contributes to the initiation and progression of multiple diseases, some of great medical relevance, such as cardiovascular and neurodegenerative diseases and cancer. In addition, thanks to the existence of the information from thousands of patient’s genomes, it has also been shown that mutations occurring in non-coding genes are key for the development and poor prognosis of many human diseases, for example, cancer [10].

## 2. Types of Non-Coding RNAs, Biogenesis and Their Cellular Functions

**Figure 1:**
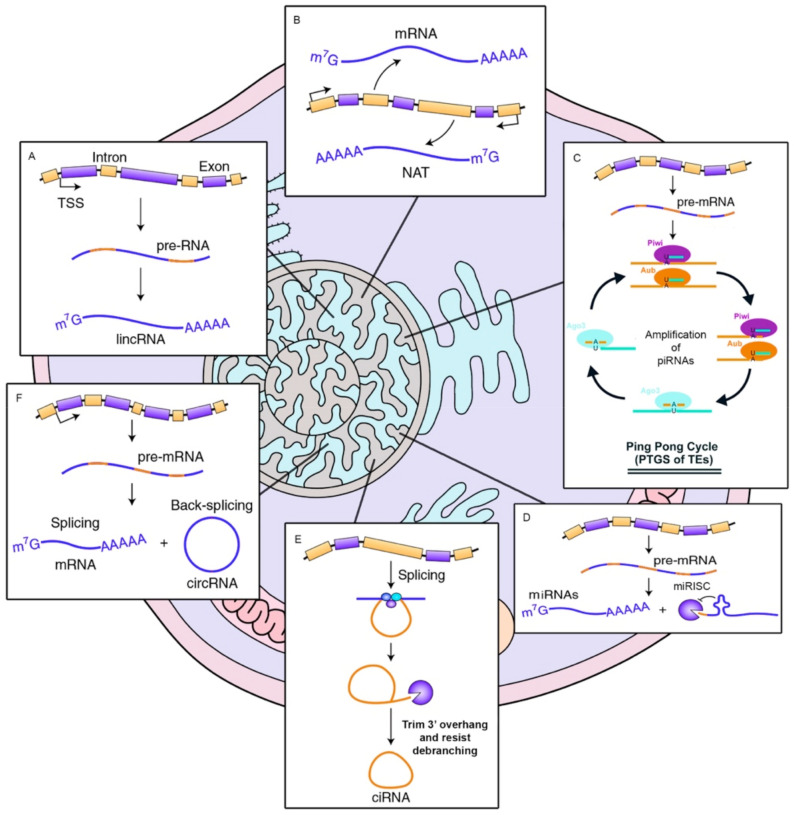
Biogenesis of ncRNAs. (**A**) long intergenic non-coding RNAs are transcribed by Pol II in a pre-RNA immature form. Once matured, they are poly-adenylated. (**B**) Natural antisense transcripts transcription from the opposed strand of protein-coding genes. (**C**) piRNAs recognize their targets and recruits piwi proteins. It results in the cleavage of the primary piRNA transcript, producing the secondary piRNA in an amplification mechanism called ping-pong. (**D**) miRNAs exported are bound with AGO proteins to form the miRISC complex to inhibit mRNA expression. (**E**,**F**) circular RNAs, (derived from lariats introns: ciRNAs, or from back-splicing events of exonic pre-mRNAs: circRNAs) are covalently closed and usually contains miRNA binding sites.

ncRNAs are usually classified in accordance with their size. For instance, short RNAs are those smaller than 200 nucleotides in length. In this group there are included small interfering RNAs (siRNAs), piwi-interacting RNAs (piRNAs), and microRNAs (miRNAs) [10,11]. On the other hand, we also find long noncoding RNAs (lncRNAs) which are longer than 200 nucleotides [12]. It is accepted that most ncRNAs are associated to gene expression regulation, but in addition, it has recently been discovered that ncRNAs are also involved in guide DNA synthesis or genome rearrangement [13]. This fact implies that these RNAs are capable of modulating both, gene production and genome reorganization [2]. To perform their role, some ncRNAs need their secondary RNA structure (such as ribozymes and riboswitches), although most of them require a complementary sequence (snRNP, snoRNP, miRNA, piRNA, and lncRNA) to accomplish its cellular role [14,15]. Please see Figure 1.

### 2.1. miRNAs

The first miRNA was discovered simultaneously in the laboratories of Dr. Victor Ambros and Dr. Gary Ruvkun in 1993, in their research on Caenorhabditis elegans [16,17]. Since then, a very abundant number of small RNAs have been discovered in hundreds of different organisms. These findings are mainly due to the emergence of new deep sequencing technologies and the development of numerous computational identification programs [18]. miRNAs are transcribed from the DNA in the form of single stranded RNAs of approximately 22 nucleotides in length. The transcription of most of the genes that produce miRNAs is mediated by RNA polymerase II (Pol II) [19,20]. In a first step, a pri-miRNA is encoded from the genomic DNA, which can be several kilobases long. This long RNA is processed by a complex constituted by the DGCR8 RNA binding protein and a ribonuclease III enzyme called Drosha, releasing a small molecule called pre-miRNA. These are driven to the cytoplasm through the Exportin 5 protein. Later, the RNAse III Dicer [21,22] proceeds to the elimination of the loop located at the end of the molecule. The disposal of the miRNA chain defines the name of its mature form; in that sense, the 5p chain emerges from the 5′ portion of the pre-miRNA fork, whereas the 3p chain derives from the 3′ side. In that sense, both mature forms bind to an Argonaute (AGO) protein to form an ATP-dependent miRNA-induced silencer complex (miRISC) [23].

In addition to this biogenesis route, commonly referred as the canonical pathway, other forms of maturation and formation of different mature miRNAs have been identified. These routes are known as non-canonical pathways and originate from the combined use of the proteins from the main pathway: Drosha, Dicer, exportin 5 and AGO2. These are mainly classified into independent Drosha/DGCR8 and independent Dicer routes. For instance, Pol III-mediated transcription of miRNAs does occur [24]. Different mechanisms have been associated to this alternative route, like how Pol III regulates the presence of cytosolic RNA:DNA hybrids binding to several components of the microRNA (miRNA) machinery, including AGO2 and DDX17, in human cell lines [25]. Moreover, specific interactions of the RNA Pol III B-box element with C/EBPβ via an embedded “C/EBPβ responsive element” (CRE) is essential for the recruitment of the RNA Pol III initiation complex and associated transcription of miRNAs (i.e., miR-138) [24]. In addition, there is a high similarity of the pre-miRNAs originated by the Drosha/DGCR8-independent pathway with Dicer substrates. For example, in plants and vertebrates appear mirtrons, originated from mRNA introns after splicing and considered as pre-miRNAs [24]. Another example of alternative pathways is the processing by Drosha of endogenous short hairpin RNA (shRNA) transcripts, as the miR-451, well conserved in vertebrates, generating Dicer-independent miRNAs [26]. To perform their cellular function, miRNAs recognize specific sequences on target mRNAs (predominantly localized within the 3′-UTR). The binding of both sequences triggers a translational inhibition that leads to a mRNA degradation [27,28]. Nevertheless, the mechanism of repression that miRNAs exert on genes does not cause enormous changes in the amount of mRNA present in the cell, which is why they are considered as fine regulators of expression, instead of being strong post-transcriptional repressors [29]. The sequence where the binding occurs between the miRNA, called seed region, and the mRNA are not 100% complementary, this is the reason why a specific miRNA is capable of regulating many mRNAs. In turn, each mRNA could be controlled by numerous miRNAs [30]. The result of this complex mechanism makes it completely difficult to decode the underlying mechanisms that elicit the cellular function of all miRNAs. In general terms, miRNAs play a fundamental function in the regulation of gene expression and monitoring various cellular processes [31]. Interestingly, miRNAs are linked to such important processes as cell differentiation [32] and disease development [33].

### 2.2. lncRNAs

Long non-coding RNAs is a broad concept that includes different RNA molecules which size is larger than 200 nucleotides. It generates a very heterogeneous class of non-coding RNAs that encompass a wide range of cellular activities, still unclear and even today is a subject of intense debate [34]. There are different types of lncRNAs according to their position in genome: those with intergenic location; that is, they are placed among genes (coding or non-coding), are known as long intergenic non-coding RNA genes (lincRNAs). Others, however, are arranged in the introns of the genes that encode proteins. In addition, there are natural antisense transcripts (NATs) originated from the opposed strand to a coding-protein gene, which implies that these RNAs have a complementary and overlapping sequence. Please see Table 2.

The biogenesis of lncRNA, is analogous to most mRNA, where RNA polymerase II (Pol II) [35] take care of the transcription; they are often 5′-capped, polyadenylated and experience alternative splicing, with an average of 2.3 isoforms per locus [36]. In most cases these lncRNAs have an unknown function and are only differentiated from the mRNAs due to the absence of a known ORF. In general, lncRNA tends to be shorter than mRNA, they have fewer number of exons, although they are larger. They are expressed at low levels and exhibit a lower conservation of the primary sequence among nearby organisms [36].

Numerous works have published a variety of different processes where lncRNAs are implicated. Among them, we highlight responses to cell stress, development, and human diseases, including cancer [37,38]. More specifically, it is thought that they are involved in an enormous number of biological activities, such as modifying gene expression (they can perform their regulatory role both in *cis*, controlling nearby genes, and in trans by altering genes separated at great distances in the genome) and transcription regulation through a variety of mechanisms: interference in polymerase activity [39], antisense RNA sequence matching [40], inhibition of histone acetyltransferase activity and repression of transcription [41], recruitment of transcriptional regulators [42], and chromatin remodeling. They can also upregulate translation of specific mRNAs without altering gene expression (SINEUPs) [43]. Certain lncRNAs have enhancer capabilities (eRNAs), as Orom et al. [44] revealed that partial elimination in the genome of lncRNAs results in a reduction in the expression levels of nearby genes. In turn, some long non-coding RNAs also host miRNA binding sites that hijack specific miRNAs in the cytoplasm and therefore avoiding the regulation of their target genes. Due to the increasing number of the proteomic studies during the last years, different “coding” competencies have been associated to lncRNAs that include short open reading frames (sORFs) sequences and are able to be translated, even though just a minority of their products will become functional and stable peptides [45,46].

In the case of NAT, both positive and negative effects have been published of their resultant sense transcription. For example, BACE1-AS is produced from the b-secretase-1 (BACE1) gene. Due to its sequence complementarity, it is able to bind to the BACE1 mRNA and safeguarding from miRNA-dependent inhibition [47]. The brain-derived neurotrophic factor (BDNF), instead of this, is usually repressed by a non-coding antisense RNA called BDNF-AS, which can bring EZH2 and PRC2 closer to the BDNF promoter region [48].

According to recent evidences, lncRNAs not only modulate protein-coding mRNA levels, like miRNAs, but also contribute to epigenetic regulation, like histone modifications and DNA methylation [49,50].

### 2.3. PiRNAs

PiRNAs are another type of non-coding RNAs with an approximate length between 23 and 32 nucleotides. Unlike the rest of small non-coding RNAs, piRNAs are originated through an independent Dicer mechanism and are generated from single-chain precursors. The processing suffered by immature piRNAs until its maturation is partially unknown. In fact, it seems that commonly recognized sequence tags that grant precursor licenses for small RNA biogenesis are missing. Therefore, any cell transcription event can unveil the theoretical creation of piRNAs, although promiscuous processing operates at a relatively low level and only a few substrates are specifically selected for an efficient piRNA biogenesis. Even though this is not well understood, several models have been proposed to explain the selection of piRNA precursors: one model favors the coupling of the transcription of piRNA precursors upon delivery to biogenesis centers (mainly in flies [51]), but other authors propose that a sequence is sufficient to trigger the processing [52].

The biogenesis of piRNAs is an event that occurs in the cytoplasm and involves several nucleases. This process is divided into two distinct branches: primary biogenesis, which acts on cluster transcripts, and the ping-pong cycle, directed towards transposon mRNAs (also called secondary biogenesis). Primary biogenesis is mediated by Zuc protein, which requires a machinery belonging to mitochondria, while the ping-pong loop depends on numerous proteins from the Tudor domain. The ping-pong loop and Zuc-dependent biogenesis are interconnected and generate piRNAs that are mostly bounded to Piwi proteins, which allows crosstalk between the cytoplasmic target and nuclear chromatin alterations.

In most species, a very small number of loci are known to be responsible for producing the vast majority of piRNAs, the so-called piRNA clusters. To date, it is known that piRNA clusters come in several configurations and are approximately divided into single chain and double chain clusters, based on their ability to generate piRNA from one or both genomic chains. In flies and mammals, piRNAs directly originated from the piRNA clusters constitute more than 90% of the piRNA population.

The previous knowledge related to the specific function of piRNA was that they interact with PIWI proteins in initial stages of embryogenesis to block transposable elements [53]. However, recently it has been discovered that piRNAs play an essential function in expression regulation through diverse processes [54]. Those piRNAs generated from primary biogenesis are processed in the cytoplasm in the form of smaller sequences. Subsequently, they interact with PIWI proteins generating the piRNA–PIWI complex, which is re-exported to the nucleus to exert its function. In this way it binds the genomic DNA through sequence complementarity, which triggers the silencing machinery and allows blocking the transcription of that particular gene. This is the general mechanism by which piRNA acts as regulators of transcription, acting primarily on transposable element sequences [55,56].

In the case of piRNAs produced by the ping-pong mechanism, these are already found in the cytoplasm where they bind to AUB or AGO3 proteins instead of coupling with PIWI proteins. In that sense, the piRNAs-Ago3 and piRNAs–AUB complexes comprise sequences that complement each other. Therefore, a piRNA–Ago complex is able to get involved into the process of maturation of RNA sequences producing a substrate for the formation of a new piRNA complex together with an AUB protein. Similarly, the formation procedure of the piRNA–Aub protein complex creates a corresponding RNA sequence substrate for the formation of new piRNA–Ago3 complexes in a process named amplification mechanism [57].

Comparable to the assembly of the miRNA silencer complex (miRISC), the piRNA silencer complex (piRISC) occurs in the cytoplasm. The piRISC safeguards the genome from external genomic sequences by blocking them.

### 2.4. circRNAs

Circular RNAs (circRNA) are single stranded RNAs capable of forming covalently closed structures, and therefore circular RNA molecules. This type of RNA is considered as non-coding RNA, however latest studies indicate that some circular RNAs are able to code for proteins [58]. The circRNAs are mainly composed of sequences that come from both exonic and intronic regions that, as a result of a subsequent splicing event [59,60], have covalently closed 5′ and 3′ ends.

Like most RNAs, circRNAs are transcribed by the enzyme RNA Pol II in the form of pre-messenger RNA (pre-mRNA) [61]. In the case of circRNAs, they suffer from a succeeding splicing event that favors the formation of a circular RNA molecule. Accordingly, if circRNAs derives from linear mRNAs, the genesis of circRNAs leads to a decrease in the number of mRNAs. This results in a negative correlation between linear and circular RNA species [61]. The circularization process is not fully elucidated but two mechanisms have been proposed where both depend on the spliceosome machinery [62]. The first suggested method occurs if a pair of downstream splice contributors, having an upstream splice acceptor not spliced, lead to the RNA to be covalently circularized. The other proposed process is called “exon omission”, it involves a splicing in loop structures (lariats) produced after an exon scape event [63].

Circular RNAs can monitor the production of linear transcripts of RNA and microRNAs and, consequently, play an important function in the regulation of gene transcription [64]. Thus, it has been confirmed that circular RNAs, as mentioned previously with lncRNAs, contain complementary sequences for miRNA. This allows circRNAs to bind miRNAs and decrease their concentration in the cytoplasm. This sponge-like system prevents miRNAs from inhibiting gene expression [65]. An example of this regulation system is found in the Sry gene, which belongs to the sex-responsible Y region. When miR-138 is highly expressed, it co-precipitates with the Argonaut 2 protein (AGO2) and with the Sry circRNA, which in its sequence contains up to 16 binding regions for miR-138. Furthermore, similar to other linear RNA transcripts that do not encode proteins, circRNAs can bind RNA binding proteins. For example, the circular transcript of the Foxo3 gene, circ-Foxo3, seems to be related to cell cycle through CDK2 and P21 interactions, which is implied in regulating and preventing anomalous proliferation [66].

In recent years, researchers have pointed out the implication of ncRNAs in high clinical impact diseases, such as cancer, neurodegenerative, and cardiovascular disorders, among others [64,67].

## 3. Involvement in Human Diseases

**Figure 2:**
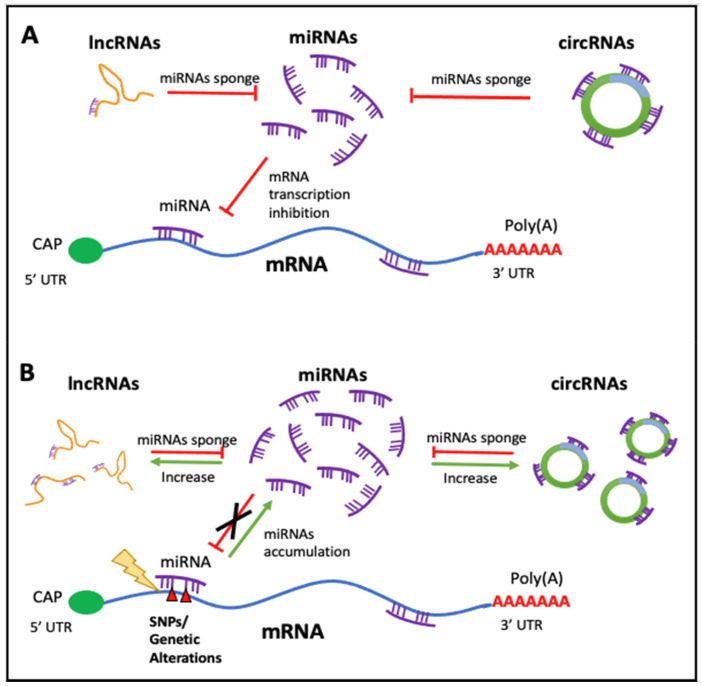
Regulatory feedback between ncRNAs. The interconnection existent among the main ncRNAs species involved in human diseases. (**A**) Regular interaction in a normal scenario (healthy cells). (**B**) When an alteration occurs (i.e., SNPs, mutation, expression alteration) there is an imbalance in the competing endogenous RNA (ceRNA) characteristic of many diseases.

There is an obvious relationship between all the ncRNAs we have described, so they should not be seen as RNAs that exert their function in isolation. As it has been aforementioned, miRNAs are negative modulators of gene expression since they bind to mRNAs of different genes (a miRNA can regulate several different mRNAs and therefore a gene can be regulated by more than one different miRNA); thus, these ncRNAs are linked to gene regulatory networks. Moreover, miRNAs functionally cooperate with ncRNA molecules, such as circRNAs and lncRNAs, to control their cellular presence. In turn, lncRNAs and circRNAs module the expression of miRNAs by a sponge-like process sharing common MRE (miRNAs responses elements), inhibiting normal miRNA targeting activity on mRNA. The alterations of this equilibrium have a very important outcome that affects the cellular fate, since important imbalances can propitiate the development of diseases. (Please see Figure 2). In consequence, this competing endogenous RNA (ceRNA) network has been found to be distorted in many diseases such as cancer [68] or cardiovascular diseases [69], among others.

Another relevant processes that affect the expression and function of ncRNAs, and thus the network of their targets, are genetic variants. These sequence changes are associated with diseases by performing genome-wide association studies (GWAS). The majority of GWAS-associated variants fall in ncRNAs loci, suggesting that they affect complex traits and diseases by altering expression of nearby genes, through regulatory mechanisms [70]. Among the examples that will be mentioned, we will see how the presence of SNPs in the sequence of a ncRNA can inhibit its maturation and therefore its cellular function. The presence of these variants in promoter regions also affects the expression levels of ncRNAs.This fact confers a higher probability of suffering from a disease. Finally, since ncRNAs exert their function by means of complementary sequence binding to their target RNAs, sequence changes in these regions (in the ncRNA itself or in its targets) can alter the binding efficiency. ln line with this, Gong et al. predicted that 52% of SNPs in the dbSNP database (release 132) would be able to create novel miRNA binding sites [71].

This review will be focused on explaining how the most described non-coding RNA species (miRNAs, lncRNAs, piRNAs, and circRNAs) play an important role in the development of human diseases. A summary of different types of ncRNAs, along with their chromosome location and their implication in various human diseases, can be found in Supplementary Table S1. Among them, we highlight the following ones due to their high incidence in the population:

### 3.1. Non-Coding RNAs in Cancer

A large number of laboratories have published and experimentally validated that alteration in the expression profiles of certain non-coding RNAs are linked to development of cancer [72]. In addition, single nucleotide polymorphisms (SNPs) are the genetic variations that are most frequently associated with the onset of cancer. Remarkably, 85% of SNPs are located in non-coding regions and linked to the development of this disease [73].

**Figure 3:**
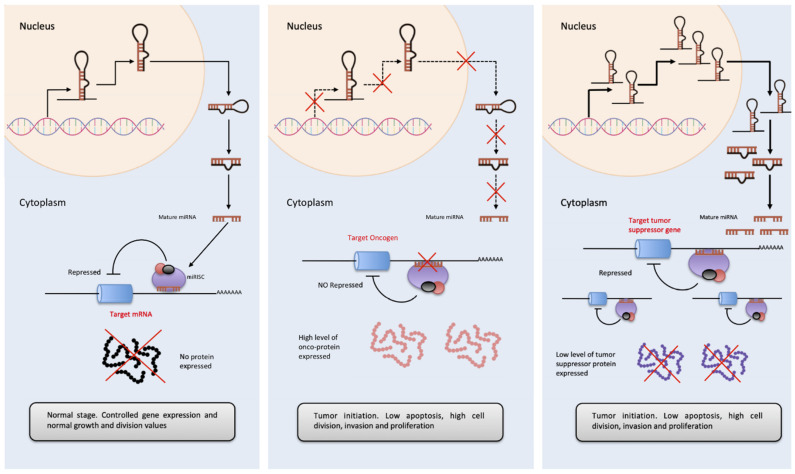
OncomiRs involvement in tumor initiation process. **Left**. The diagram represents basal expression of miRNAs. These are released to the cytoplasm in a highly controlled amount, to exert their function by binding to the 3′-UTR of their target mRNA, inhibiting the formation of protein. **Center**. In the case of cancer, the expression of multiple genes is altered, if this variation causes an inhibition of OncomiRs (miRNAs that regulate oncogene expressions), these are not able to inhibit the amount of oncogenic protein, favoring proliferation, cell cycle progression as well as a decrease in apoptosis and in the tumor initiation. **Right**. On the contrary, in cancer, the overexpression of miRNAs acting as tumor suppressor regulators has also been observed (essentially, they are miRNAs that inhibit the expression of tumor suppressor genes). A considerable increase in the cytoplasm of OncomiRs, causes the almost disappearance of tumor suppressor proteins, favoring the development of cancer, its progression and tumor invasion.

In the case of miRNA and more specifically in cancer, there are the so-called OncomiRs, which appear overexpressed in tumor samples and linked to tumor development [74]. It has been reported that OncomiRs affect the proliferation and signaling of cancer cells, prevent apoptosis, favors invasion, angiogenesis and metastasis [75]. (Please see Figure 3.) For example, the miR-17/92 cluster was the first OncomiR described. This is a polycistronic RNA that encodes for 6 miRNAs. Its overexpression is related to the inhibition of E2F1 [76] and AIB1 genes [77] triggering tumor initiation by stimulating cell progression and proliferation. On the contrary, anti-OncomiRs are a group of miRNAs that behaves as tumor suppressors, such as the miR-143/145 cluster. Both miRNAs control the expression of dozens of genes and its deregulation is believed to propitiate the appearance of the initial events in cancer. In comparison with cells from non-tumor tissues, these miRNAs are expressed in a smaller amount in colon, head and neck, breast, bladder, and lung cancers [78].

This indicates that a recurrent oncogenic process is implicated. Well known is the TP53 gene loss-of-function, as the activation of the p53 pathway increases the miR-143 and miR-145 levels through a transcriptional mechanism [79]. Another mechanism that affects miR-143 and miR-145 levels depends on the mitogen-activated protein kinase (MAPK) cascade activity, with RREB1 being the effector for the repression of the cluster [80]. In addition, in the case of colorectal cancer, the variant rs353292 in the flanking region of miR-143/145 increases the risk of developing this disease [81], as rs353292 CT/TT individuals presented a lower expression of miR-143.

Furthermore, there is a group of miRNAs involved in an extensive range of diseases, those related to hypoxia (low oxygen levels) and called hypoxamirs. In the case of cancer, there are a high number of tumors with hypoxic tendency (being large masses of tissue with a high proliferation rate and little vascular development, the development of hypoxic regions is favored), such as breast cancer. Among this group of microRNAs, we must highlight miR-210, recognized as a mediator of cellular responses to stress related to hypoxia. Recent studies have postulated that hypoxamirs regulate hypoxic transcriptional cascades, angiogenesis, and endothelial growth [82] and consequently, involved in metastasis. miRNAs that suffer an alteration in their expression levels, have been related to various functions in tumoral events, including the maintenance of proliferation, resistance to cell death, invasion, and metastasis and very recently with resistance to drugs [83]. These anomalous expression patterns have been endorsed to DNA modifications (deletions, amplifications, or mutations) [84], epigenetic alterations [85], miss-regulated transcription factors [86], and deregulation of RNA-binding proteins (RBP) that contribute to miRNA synthesis [87].

In addition, single nucleotide polymorphisms (SNPs) are the genetic variations that are most frequently associated with the onset of cancer. Remarkably, 85% of SNPs are located in non-coding regions and linked to the development of this disease [73]. For example, the SNP rs11671784 is localized within the miR-27a sequence affecting its processing efficiency. This variant has been implicated in gastric cancer reduction risk by impairing the maturation of pre-miR-27a to mature miR-27a [88].

According to the atlas of human long non-coding RNAs in FANTOM5 [89], there are about 28,000 known human lncRNA genes, making them the most abundant transcribed RNAs from DNA. They can work as enhancers, scaffolds or decoys by binding with RNAs or even with RNA binding proteins, which alters all cellular signaling regulation networks [2]. For instance, the lncRNA MEG3 (maternally expressed gene 3) binds the p53 DNA binding domain, favoring the activation of P53 [90]. Interestingly, in cervical cancer, MEG3 expression is inversely associated to tumor volume and metastasis, which indicates its strong role as a tumor suppressor through P53 and pointing out as a possible therapeutic target [91]. Likewise, low levels of MEG3 have been linked with an increment in cell cycle progression and autophagy in bladder cancer [92]. Moreover, another example of the role of the long non-coding RNAs in gene regulation is the lncRNA-PVT1 that usually acts as miRNA sponge towards the miR-200 family in normal breast tissue and loses its functionality in breast cancer cells, altering the genetic regulation in this tissue [93].

A recent study proved that LINC00883 could act as miRNA “sponge” for miR-150 [94] which promotes tumor cell proliferation by negatively regulating tumor suppressor gene SRCIN1 [95] and Notch3 [96]. In fact, Notch3 played an important role in oncogenesis and resistance to chemotherapy in lung cancer [97]. Qi Sun et al. demonstrated that T allele of rs793544 was associated with the low expression level of LINC00883 in lung tumor tissues [98]. Taken together, it seems that SNP rs793544 is related with the diminished expression level of LNC00883, increasing the risk of lung cancer by altering the expression of miR-150.

In the case of piRNAs, it was initially thought that their only function was related to the suppression of the activity of mobile elements (or transposons). In fact, the inhibition of piRNAs that normally suppresses the function of these elements can facilitate mutagenic retro-transpositions and DNA instability, thus favoring cancer initiation. Nevertheless, it is not an exclusive mechanism, because it has been proposed that the piRNA/PIWI complex can cause aberrant DNA methylation, which results in genomic silencing, stimulating the cell to enter in a “stem-like” stage [99]. In detail, piRNAs can stimulate *cis* methylation, in human lymphoma and breast cancer cell lines, single copy piRNAs favor methylation of gene-specific DNA through imperfect binding to genomic DNA [100]. Moreover, piRNAs have been associated to cell cycle regulation, in fact, it has been discovered that alteration in the expression of piRNA/PIWI complex correlates with clinical variables in tumorigenic samples indicating a new role for piRNA in cancer. For instance, in breast cancer, four significantly regulated piRNAs have been discovered: piR-20365, piR-4987, piR-20582, and piR-20485 [101]. In addition, genetic association studies have identified variants associated with cancer in piRNA loci. As an example, the SNP rs147061479 in piR-598 increases glioma risk by affecting the tumor-suppressive function of piR-598 [102].

Finally, as we mentioned, miRNAs are crucial key actors in the pathogenesis of most human tumors [103]. Therefore, because circRNAs function as miRNA modulators, these in turn are also linked to cancer. Currently, a small number of circRNAs have been discovered with several targets’ sites for a particular miRNA, but it has also been observed that most of the circRNAs have, in fact, other functions besides the regulation of miRNA [104]. For example, it has been revealed that circRNAs are highly expressed in several tumor cell lines from the ENCODE consortium data [105]. These studies have demonstrated the involvement of circRNAs in cancer through unexpected functions. This is the case of colorectal carcinoma (RCC) in which Bachmayr-Heyda et al. confirmed a general decrease in circRNAs expression levels in tumor tissue compared to adjacent non-cancerous tissues [106]. This reduction has been negatively correlated with proliferation. The authors affirmed that circRNAs are accumulated in stable-arrested cells, while they are disseminated among the proliferating ones.

### 3.2. Neurodegenerative Diseases

Although ncRNAs are expressed in all cell types, the Central Nervous System (CNS) is especially enriched [107]. Thus, approximately 40% of the genes encoding lncRNA are specifically expressed in brain tissue. Other types of non-coding RNAs, such as circRNA and certain miRNAs, are also specifically abundant in the CNS (and some of them specifically involved in synapses) [108,109]. miRNAs such as miR-124 and miR-132 have a regulatory impact on neurogenesis [110], while other lncRNAs, as rhab-domyosarcoma 2 associated transcript (RMST) and Tcl1 upstream neuron-associated long intergenic ncRNA (TUNA), stimulates neuronal differentiation [111]. In addition, ncRNAs enriched in synaptic connections are known, for example certain miRNA precursors and several miRNAs (e.g., miR-9, miR-132, miR-134, and miR-138) [112]. Moreover, most circRNAs that are expressed in the brain act as synaptic regulators to regulate local protein expression.

In the case of Parkinson Disease (PD), it has been found that there is a negative correlation between the levels of expression of certain miRNAs and two of the genes involved in this disease: α-synuclein (SNCA) and leucine-rich repeat kinase2 (LRRK2). For instance, miR-7 and miR-153 [113] are natural regulators of SNCA and LRRK2. Both miRNAs are highly expressed in the brain [114]. In addition, a specific circRNA related to this disease has also been found through the inhibition of miR-7. Recent research has revealed that circRNA CDR1 acts as a negative regulator of miR-7 causing increased expression of SNCA, which is involved in the development of PD and contributes to oxidative stress. Therefore, circRNA CDR1AS as a miR-7 sponge plays a crucial role in PD by repressing miR-7 [61].

GWAS studies in PD cohorts have revealed that polymorphisms such as rs2070535 and rs10849446 are located in genes highly related to PD, as PDCK and SCNN1A [115,116]. Further studies on these polymorphisms have determined that rs2070535 is located in the 3′-UTR region of PDCK, and differential expression of miRNAs may influence the changes in expression of this gene as seen in this disease. Similarly, rs10849446 is located in a intronic region of SCNN1A related to lncRNAs [117].

LncRNAs involved in PD have also been discovered, for example the antisense transcript of the UCHL1 gene (UCHL1-AS) is also linked to the prognosis of this disease. The Ubiquitin carboxyl-terminal hydrolase isozyme L1 (UCHL1] is highly expressed in the substantia nigra and related to neuron differentiation. Several authors had pointed out that UCHL1 is a PD risk gene [118]. In that sense, UCHL1-AS targets Uchl1 mRNA to induce its translation and increasing UCHL1 levels [43].

Alzheimer’s disease (AD) is another type of neurodegenerative disorder caused by many different factors. Among them, we can highlight the following ones related to ncRNAs. Elevated levels of amyloid-β protein (Aβ), BACE1 proteins, as well as the NAT generated in the strand opposite to this gene, BACE1-AS, have been detected in AD. Aβ is resulting from the cleavage of the amyloid precursor protein (APP) by the APP enzyme of the beta 1 site (BACE1) and the γ-secretase complex. The aberrant expression of these molecules has been related with numerous neurological syndromes and mainly in subjects with Alzheimer’s disease, which emphasizes the importance of regulating the catalytic activity of BACE1 in this disease. In that sense, it was discovered that the CIRS-7 circRNA, downregulated in AD brain tissue, has the main function of reducing APP and BACE1 protein levels [119]. In addition, other set of circRNAs has been implicated in AD through the alteration of myelin function [120].

Another example is 17A, this is a ncRNA of a size of 159 nt from the third intron of the G 51 gene receptor coupled to the G protein (GPR51). This ncRNA is involved in increasing expression of the GABA B receptor affecting all signaling cascades dependent on GABA B. ncRNA 17A is overexpressed in AD patients versus healthy tissues, proposing that it might be involved somehow in the initiation of AD [121,122].

Furthermore, miRNAs are also associated in the deposition of Aβ, the formation of neurofibrillar nodes (NFT) and extensive neuronal degeneration in the brain. It has been found that miRNAs are able to control APP expression in several ways: for example, miR-106a, miR-520c, members of the miR-20a family (miR-20a, miR-17), miR-16, miR-101, miR-147, miR-655, miR-323-3p, miR-644, and miR-153 are able to bind to a specific sequence in the 3′UTR of APP. Through experimental in vitro studies, the reduction in the expression level of APP when co-expressed with these miRNAs has been verified [123,124].

Single nucleotide polymorphisms in ncRNAs have been also associated with AD. It is important to mention that rs7232 and rs12453 in lncRNA NONHSAT160355.1 significantly downregulate the expression of a known AD pathogenic gene, TCN1 in Temporal Cortex, which participates in the regulation of homocysteine in brain to increase risk of AD [125].

#### 3.2.1. Cardiovascular Diseases

Cardiovascular diseases (CVD) are considered the first cause of mortality all over the world. This concept involves not only heart diseases but also blood vessel associated diseases. The main risk factors classically associated with these diseases are hypertension, diabetes, and smoking, and can be used for stratification and prediction factors for prognosis of the patients. The rest of the risk factors can vary depending on the pathology examined.

Due to the high number of diseases associated to this term, in this section we are going to examine the main ncRNAs historically associated with the development of CVD, and two different cardiovascular pathologies such as atherosclerosis and myocardial infarction.

The lncRNA Braveheart (lncRNA-Bvhrt) was the first one described with a functional implication in cardiac function in mouse heart development. This lncRNA has been associated to the cardiac linage establishment during cardiac cells differentiation [126]. This role was performed through the interactions with other gene implicated in cell differentiation, SUZ12 (part of the polycomb-repressive complex 2, PRC2), pointing out to the possible role of lncRNA-Bvht in the epigenetic regulation of cardiac commitment [127]. Furthermore, it suggested the implication of lncRNA-Bvht in directing the neonatal Cardiomyocytes (CM) to a cardiac differentiation. It has been described its involvement in mouse stem cells differentiation to CMs. Other lncRNA called lncRNA-fendrr was described as a proper modulator of heart differentiation at chromatin level [128].

The implication of other ncRNA, lncRNA-HBL1 (Heart Break LncRNA1) was recently unveil in human induced pluripotent stem cells (hiPSCs) acting as a regulator of CM development. The overexpression of this lncRNA inhibit the CM differentiation from hiPSCs. This is not a direct procedure due to the mechanism of sequestration of hsa-miR-1 during the process. The deregulation of the expression of lncRNA-HBL1 has been described in ischemic heart failure and several cardiovascular diseases in mouse models and human [129]. Moreover, every cardiac pathophysiology has some lncRNAs which are different for the particular cell type related to each condition.

In contrast with other diseases, in cardiovascular diseases several lncRNAs have been described associated to their development and because of their potential use as a biomarker. In recent studies, some specific lncRNAs were associated and experimentally validated with CVDs: lncRNA ANRIL (antisense noncoding RNA located in INK4 locus), MALAT1 (metastasis associated lung adenocarcinomas transcript 1), KCNQ1OT1 (KCNQ1 overlapping transcript 1), aHIF (natural antisense transcript derived from HIF1alpha), and MIAT (myocardial infarction associated transcript). In that sense, the 9p21.3 risk locus, identified in several genome-wide association studies (GWAS) for coronary artery disease (CAD) susceptibility, is adjacent to the last exons of ANRIL which encompasses multiple SNPs [130].

The potential therapeutic application of these lncRNAs in atherosclerosis was pointed out since the expression levels of MIAT and ANRIL were higher compared with a non-atherosclerotic tissue control, as well as the expression levels of MALAT were lower than control [131].

Other classes of ncRNAs, as miRNAs, have been also linked to the development of cardiovascular diseases. Several miRNAs currently related with cardiovascular disorders were previously described as onco-miRs, in association with different cancer types, or related with other cellular functions that are not directly associated with CADs. Some miRNAs like miR-126-3p or miR-21-5p play a key role in cardiovascular diseases such as the response to ischemia process, regulating myocardial fibrosis, ventricular remodeling, arrythmia, or heart failure. In a similar manner, rs687289-A and rs532436-A are intronic variants to the ABO gene relevant to thrombosis and atherosclerosis risk. Nikpay M. et al. stated that rs532436-A and rs687289-A are associated to an increase level of miR-10b-5p, and higher circulating level of miR-10b-5p is associated with increased risk of CAD [132].

Recently, it was published the updated version of HMDD2.0 database [133]. This database included experimentally validated associations between miRNAs and different diseases, and included 165 cardiovascular-related miRNAs, spread across the human genome.

Within the definition of Cardiovascular diseases, atherosclerosis is one of the main concerns in the field. It has been defined as a multifactorial disease that involves multiple associated mechanisms, traditionally described as a chronic inflammatory and lipid disorder, and implicating diverse cell types [134]. During the study of atherosclerosis and its vascular-associated complications, different evidences have shown the involvement of ncRNAs in the development of this disease, and it is considered as other epigenetic mechanism implicated. Even more, the epigenome-wide associated studies allowed to observe the underlying implication of the epigenetic regulation of several related functions with the development of atherosclerosis, such as inflammation, lipid metabolism, and redox cellular status.

Maegdefesset and colleagues were investigating the advantages of a local delivery of some mimic miRs to increase the expression of miR-21 and miR-210 [135,136]. Replacing the lack of these miRNAs, conferred stability to the atherosclerotic plaque, decreasing the risk of atherothrombotic vascular complications. Recent studies found a useful signature of five plasmatic miRNAs (circulating miRNAs) for predicting Myocardial Infarction (MI) with a percentage of accuracy of the 81.8% in women and the 74.1% in men. This panel of circulating miRNAs was generated using the data extracted from the HUNT study [137] and included the following miRNAs: let-7g-5p, miR-106a-5p, miR-424-5p, miR-144-3p, and miR-660-5p. Other circulating miRNAs, which expression is p53-dependent, were described as a novel predictor of heart failure after suffered from MI: miR-192-5p, miR-194-5p, and miR-34a-5p [138]. General speaking, there is more than 60 miRNAs already associated with CVDs that could be used with diagnostic and prognostic purposes.

The association of another circulating lncRNAs, Zinc finger antisense 1 (ZFAS1) and CDR1 antisense (CDR1AS), has been described as differentially expressed in acute MI patients in comparison with healthy individuals [139]. The urothelial carcinoma-associated 1 (UCA1) is a lncRNA that was previously associated to bladder and lung cancer, and it was known as a predictive biomarker for its expression in this kind of cancers. Furthermore, it was described its expression in heart tissue of healthy individuals. Recent findings have shown its deregulation from early stage of acute MI until three days after the MI. Moreover, there is an inverse correlating among circulating UCA1 levels and miR-1 expression levels [140].

The presence of circRNAs in human heart tissue is directly related with the abundance of their related mRNAs. Some of the most important cardiac expressed transcript, as RYR2, DMD, and TTN genes, generate the most abundant circRNAs in heart tissue. In atherosclerosis, CDKN2B-AS1 is perhaps one of the molecularly best-studied circRNAs. According to other studies, this circular RNA from the 9p21 locus, contains a vast number of single nucleotide polymorphisms that have been linked to atherosclerotic vascular disease, as well as to type 2 diabetes mellitus (T2DM) [141].

#### 3.2.2. Other Diseases

The purpose of this section is to include some of the remaining diseases that could obtain a benefit from the research in ncRNAs as a potential therapeutic target, as well as biomarkers for their use in diagnosis and prognosis of the different pathologies.

Type 1 diabetes is a chronic disease in which very low or absent insulin production by the pancreas has two main consequences: insulitis and pancreatic beta cells destruction. In the organism response to this kind of stress, the pro-inflammatory cytokines interferon gamma (IFNG) and interleukin 1 beta (IL1B) are produced during insulitis and enhance endoplasmic reticulum (ER) stress response and trigger the expression of BCL2-related proteins in beta cells, participating in their cell death. Grieco et al., has recently unveil the implication of the downregulation of two miRNAs associated to these facts from the same miRNA family, miR-204-5p, and miR-211-5p [142]. These miRNAs have been involved in the regulation of the transcriptional expression of some ER stress genes downstream PERK, specifically DDIT3 (CHOP, pro-apoptotic protein). These novel findings related with early type 1 diabetes point out a relationship between miRNAs, ER stress and beta cell apoptosis.

Type 2 diabetes is a form of the disease characterized by three different conditions in the human body: high levels of glucose in blood, a decrease in the amount of insulin, and an insulin resistance. This is another kind of defect of the pancreas and it is caused by an impairment between the insulin sensitivity and the insulin secretion.

The involvement of miRNAs in the production of insulin has been extensively studied. miRNAs play an important role in the regulation of the gene coding for the insulin. The expression of several miRNAs has been associated with this process. For instance, the upregulation of miR-30d directly correlated with high glucose levels. This fact has been previously described by Tang et al., enhancing the transcription of the insulin gene, and the opposite effect was observed during its inhibition [143]. Another important miRNA in the pancreas is miR-375. Its overexpression is able to inhibit the glucose-stimulate insulin expression. This miRNA achieves this effect by means of the regulation of 3′-phosphoinositide-dependent protein kinase-1 (PDK1), unveiling its important role within the pancreas [144,145,146].

The main organs depending on the glucose consumption in the organism are the liver and the skeletal muscle. Recent studies have also described the implication of other miRNAs in mechanisms associated to insulin resistance. For example, miR-29 has been described by Zhou et al., in the insulin resistance by targeting PPARδ in skeletal muscle [147]. In mice, the upregulation of miR-29 was also related with the inhibition of the gluconeogenesis. In mice liver, miR-33 regulates the expression of insulin receptor substrate 2 (IRS2), that is related with insulin resistance [148].

There are many renal diseases associated with the dysregulation of ncRNAs, specifically miRNAs, such as acute renal damage, renal cell carcinoma, diabetic nephropathy, polycystic kidney disease (PKD), and others. In the development of these diseases, it has been described an important involvement of miRNAs. Different studies were coincident in the importance of miRNAs and their use as biomarkers for the diagnosis of these renal diseases. The miR-200 family has been described as abundant in renal tissue and highly expressed [149]. Some of the components of this family has been found in a very low expression in patients suffering from IgA neuropathy, being the level of decrease of the expression considered as a prognostic factor in this disease. Moreover, the increase on the expression levels of miR-155 and miR-146a has been also correlated with a poor prognosis of the disease, at the clinical and pathophysiology level [150].

The deregulation of the expression of miRNAs can be used as a diagnostic or prognostic factor for several diseases, such as cancer. In this specific case, they could be useful in the detection of diseases related with the presence of cysts, like PKD, or associated with a process of cystogenesis in the organs with like liver, pancreas, and ovary. Thus, these miRNAs could be used as a novel biomarker for detecting renal diseases.

In the case of autoimmune diseases, there are also ncRNAs implicated at different levels. As an example, rs57095329 is located in the miR-146a promoter which confers risk of systemic lupus erythematosus (SLE). It turns out that individuals carrying the risk allele in the promoter showed lower expression levels of miR-146a [151]. Further, in SLE, the over expression of linc00513 plays a role in lupus pathogenesis by promoting IFN signalling pathway. SNP variants in the linc00513 promoter are functionally significant in regulating linc00513 expression and conferring predisposition to SLE [152].

## 4. Bioinformatics Approaches for Identification of Novel Targets in Diseases

The generation of a high amount of genomic high-throughput data has elucidated the necessity to develop new more powerful tools, not only for their analysis but also for storing and integrating different kind of data, proceeding from diverse sources. The alteration of a single genomic product does not use to be the cause of a disease phenotype. The consequence of that alteration over the genetic and genomic regulation and the related networks, is the main focus of study in the medical genomic landscape.

To do so, it is important to have a source of data as well as efficient and accurate tools for unveiling the possible interactions at the regulatory level. Moreover, it is essential do not forget about the accuracy of the in silico predictions and the value of always experimentally validate the proposed candidates. With this purpose, several databases have been created with empirically supported data that provide a valuable source of information. This is the case of TarBase and miRecords, in the context of RNA network interactions (RNA-RNA or RNA-DNA), that provide information about microRNA-gene targets interactions [153]. In this sense, a very helpful database that recapitulates functional genomic data focused on post-transcriptional regulation is called POSTAR2 [154]. This database includes more than 1200 CLIP-seq datasets from six different species: human, mouse, fly, worm, *Arabidopsis*, and yeast. Its new release, POSTAR3 (tsinghua.edu.cn (accessed on 14 February 2021)), includes 2075 public CLIP-seq datasets and also adds to the previous species examined, Zebrafish.

In that sense, there are a large number of repositories that store very relevant information about ncRNAs and that can help to deal with the integration of data from different sources to favor the discovery of new functions or new pathogenic mechanisms. In the field of miRNAs one of the most important resource is miRBase [155] as it stores all known information from a total of 38,589 miRNAs of 271 different organisms. In a very similar way, we can find lncRNAdb 2.0 dedicated to the study of lncRNAs [156]. This is a manually curated database storing lncRNAs from 287 eukaryotic organisms. In the case of circRNAs, circBase [157] is the reference database and contains circRNAs identified in various experiments from a total of six organisms. Finally, piRNABank [158] provides comprehensive information on piRNAs in the four most widely studied organisms: Human, Mouse, Rat, and *Drosophila*.

Regarding the relationship between ncRNAs and human diseases, few general databases can be found. We could stand out NPInter [159], that documents experimentally validated interactions between noncoding RNAs and proteins, mRNAs or genomic DNAs. Another data base of interest is called HDncRNA [160], although this database is specifically focused on the study of heart diseases.

On the other hand, we do find databases of miRNAs, lncRNAs, circRNAs, and piwis that separately document their involvement in relevant diseases. Briefly, in the case of miRNAs it is important to spotlight some relevant databases such as HMDD 3.0 [161] that manually collects a significant number of miRNA–disease association entries from literature. Moreover, miR2Disease [162] is a manually curated database of microRNA deregulation in various human diseases, as well as PhenomiR [163] that provides information about the deregulation of microRNA expression in diseases and biological processes. To this end, it is worth nothing two miRNAs databases related with cancer, OMCD (OncoMir Cancer Database) based on miRNAs expression sequencing data, derived for over 10,000 cancer patients, in which you the relevant clinical information associated to the profiles can be found, as well as organ specific controls (from The Cancer Atlas Database) [164]; and miRCancer (microRNA Cancer Association Database) that provides a collection of miRNAs expression profiles from different cancer types, extracted from literature using text mining techniques, manually curated [165].

Similarly, in the case of lncRNAs, repositories such as LncRNADisease 2.0 [166] (a database of experimentally validated and predicted lncRNAS-disease associations) and Lnc2Cancer 2.0 [167] (a manually curated database of cancer-associated lncRNAs with experimental support) have been recently created.

In turn, there are also several repositories that accumulate information about the implication of circRNAs in the development or evolution of several human diseases. Among them, it is worth noting Circ2Disease [168] (a database that contains manually curated associations between circRNAs and human diseases with strong experimental evidences), CSCD [169] (a database for cancer-specific circular RNAs) or Circ2Traits [170] (a comprehensive database for circular RNA potentially associated with disease and traits). These are the most commonly used in the field of circRNAs.

Finally, in the case of piwis, we found a useful database that focuses on the relationship between PIWI-interacting RNAs and human diseases: piRDisease v1.0 [171], a manually curated database for piRNA associated diseases.

As discussed in previous sections, numerous studies indicate that single nucleotide polymorphisms (SNPs) could contribute to diseases or traits by influencing ncRNA expression. Indeed, most of the risk SNPs detected by GWAS are located in non-coding genomic regions [172], indicating that ncRNAs may be potential causal targets of some disease-related GWAS loci. There are databases, such as ncRNA-eQTL [173], which perform genome-wide expression quantitative trait loci (eQTL) analyses to assess the effects of SNPs on ncRNA expression in cancer to decipher how risk alleles contribute to tumorigenesis. Similarly, LincSNP 3.0 [174] aims to annotate disease or phenotype associated variants in human long non-coding RNAs (lncRNAs) and circular RNAs (circRNAs) or their regulatory elements.

Related to these databases and the identification of novel druggable targets, there are two notable databases: Open Targets Genetics and DisGeNET. The former is a resource that aggregates human GWAS and functional genomics data, including gene expression, protein abundance, chromatin interaction/conformation, allowing strong connections to be inferred between GWAS-associated loci, variants, and likely causal genes/ncRNAs. A really interesting point of this portal is the possibility to prioritize genes/ncRNAs underlying disease causation and the inclusion of tools to identify new targets for drug discovery and drug repurposing [70]. In line with this, DisGeNET integrates data on disease-associated genes/ncRNAs and variants from multiple sources, including the scientific literature. It retrieves all pathways for a particular disease, or searches for disease-associated proteins that are also drug targets [175].

The development of more reliable bioinformatic tools that allow to make this kind of association between not only genomic data but also with proteomic and metabolomic data, is needed.

For instance, there is a novel bioinformatic tool that could help to predict lncRNA and disease associations (e.g., LncDisease), that has been developed by Cui Lab (http://www.cuilab.cn/ (accessed on 14 February 2021)) [176]. This could be useful for uncovering the biological functions of lncRNAs and their involvement in human diseases.

Another novel and useful tool called MiARma-Seq [18,60] (miRNA-Seq And RNA-Seq Multiprocess Analysis) has been generated for the detection of mRNAs, miRNAs, and circRNAs, to perform differential expression analysis of the data, to make an accurate target prediction and a functional analysis in transcriptome samples, avoiding a tedious software installation and/or configuration. This tool also offers a quick execution of the analyses and allows to make the analysis in a standard computer, as well as consistency of the results.

The majority of the public standard tools used up to date are included in order to perform all the calculations in a reproducible manner. The associations performed by these bioinformatic tools, although they are based in experimental data most of them coming from human samples or experimental mouse/rat models, are predictions that need to be confirmed by an experimental validation.

The existence of contradictory information in ncRNAs reports highlight the necessity of the functional validation of the bioinformatic prediction and not only the assumption of the biological roles of ncRNAs based on that unique source of information. Further exploration and improvement of the current information and methodology are needed for elucidating the involvement of ncRNAs in human diseases, and to explode their utility in the clinic as a diagnostic and prognostic biomarker in human diseases.

## 5. Importance and Future Perspectives

Since the discovery of the ncRNAs, in the last decades all the researchers’ efforts have been focused in unveil the diverse mechanisms of gene expression regulation at a human genome level, and their implication in human development and diseases.

To date, most of the exploratory work with these ncRNAs (miRNAs, lncRNAs, and more recently circRNAs and piRNAs) is dedicated to unveiling the potential utility within the human health. The most likely use of them is as specific molecular biomarkers for diagnosis and stratification of the human diseases. Because of their properties, they fit well with the features that a good biomarker should have, such as present a stable structure, being accessible using a non-invasive method of extraction, have an early presence of the signal prior to the symptomology of the disease, and a low cost of the detection methods.

Nowadays, the common biomarkers used in clinical practice are known proteins, but the detection of these molecules in blood samples, for example, sometimes represent a challenge due to the post-transcriptional modifications suffered in many cases or the low quality and quantity of the material extracted from body fluids. These are the main reasons why scientists are looking for alternatives that could improve the accuracy of the diagnostic and increase the sensitivity and the specificity of the tests applied.

Recent findings based on the characterization of extracellular RNAs has increased and become very frequent in the last decade and has given strong evidence of the presence of circulating and exosome miRNAs in several body fluids, blood, serum, plasma, saliva, urine, bile, peritoneal fluid, and cerebrospinal fluid. They seem to be very stable in these fluids and suitable for their use as a biomarkers and treatment response.

These discoveries have opened a new path to the study not only the functions of miRNAs in the body fluids and their potential use of diagnostic and prognostic tool, but also to the research of circulating lncRNAs and circRNAs as a potential biomarker for diverse diseases and even for therapeutic purposes.

It is possible that the use of mRNA-based vaccines, such as the one recently developed for covid-19, could encourage the expansion of a field that has yet to take off, and that is the therapeutic use of non-coding RNAs. The basic principle for the potential use of these RNAs for therapeutic purposes is based on the interference pathway where these ncRNAs, after binding to their targets, would fostering their degradation. The pleiotropic nature makes them interesting as a drug targets for complex untreated diseases. In fact, the first small interference RNA (siRNA) was approved by the FDA (Food and Drug administration) not long ago, in 2018. This siRNA, called Patisiran, binds to the transthyretin mRNA, causing it to be eliminated in the cytoplasm in those patients suffering from a rare disease called transthyretin-mediated hereditary amyloidosis (hATTR) [177,178].

To date, several clinical studies have been completed on miRNA biomarkers, this includes phase 4 clinical trials to identify miRNAs as biomarkers of disease progression in patients receiving FDA-approved drugs. Although no miRNA drug has been recorded into the clinicaltrials.gov database for phase 3 trials, currently miRNA-imitators or inhibitors are under study to address different disorder, highlighting the flexibility of this procedure. For Parkinson disease, let-7 appears to be a promising “drug” that acts against multiple underlying disease mechanisms [179]. Other examples of miRNA-based therapies in clinical trials are hepatitis C (AntimiR-122), type 2 diabetes and non-alcoholic fatty liver diseases (AntimiR-103/107), lymphoma and T-cell leukemias, mycosis fungoides (AntimiR-155), scleroderma (miR-29 mimicry), mesothelioma and lung cancer (miR-16 mimic), wound healing and heart failure (miR-92), keloids and fibrous scar tissue formation (miR-92), and Alport syndrome (miR-21) [180,181,182,183]. Recently, the first miRNA-based inhibitory therapeutic strategies have been tested in patients with heart failure, as well as in healthy volunteers, to study the effects on wound healing (clinical trial codes: NCT04045405; NCT03603431).

In the case of lncRNAs, most studies are still in preclinical phase. Nonetheless research on mitochondrial lncRNAs (mtlncRNA) has experience enormous progress. When mitochondrial DNA is transcribed, during transcription processing, it generates large non-coding sequences, including mtlncRNA [184], and sense (SncmtRNA) along with antisense mtlncRNA (ASncmtRNA) [185]. ASncmtRNA-1 and ASncmtRNA-2 are down-regulated in various tumor tissues [186] and it has been proposed that antisense nucleotides (ASOs) targeting the remaining few copies of ASncmtRNA, essential for cell survival, induce cell cycle arrest, and later apoptosis specifically in cancer cells. For this reason, the FDA has approved a clinical trial of Andes-1537, a short single-stranded phosphorothioate ASO that specifically binds to ASncmtRNAs, including a Phase I clinical trial recruiting patients with advanced metastatic cancer (NCT02508441) or multiple solid tumors (NCT03985072).

There is currently no piRNA-based clinical trial, as the study as possible biomarkers has barely begun. Gliomas is the disease in which most progress has been made [102,187].

Finally, there are advanced studies, including patents granted for the study of circRNAs in different diseases. For instance, patents EP3054017 A1 and WO2016124655 A1 reported 16 circRNA transcripts that are correlated with CVD, in particular, acute myocardial infarction [188,189].

The unexpected use of the RNA-technology for beating Covid-19 virus has focused the attention of the scientific community on the study of ncRNAs as transcriptional modulators, able to restore altered gene expression in different diseases. The demonstrated efficacy and biosafety of the Covid-19 vaccine and its approval by the medical authorities has opened a path for the use of RNA-based therapies in human diseases. During the following years, we may experience a significant increase in the use of this technology, and it could give rise to new promising treatments, as well as an increase and evolution of the knowledge in ncRNA field.

## Figures and Tables

**Table 1:** Types of ncRNAs and their known functions.

ncRNA Species	General Functions and Properties
miRNA	Fine regulators of expression
	Inhibition of gene transcription
lncRNA	Interference in polymerase activity
	Antisense RNA sequence matching
	Inhibition of histone acetyltransferase activity and repression of transcription
	Recruitment of transcriptional regulators
	Upregulate translation without altering mRNA levels
	Chromatin remodeling
	Enhancer
	Histone modifications and DNA methylation
piRNA	Block transposable elements
	Regulators of transcription acting primarily on transposable element sequences
	Protection against external nucleic acids
circRNA	Monitor the production of linear transcripts of RNA and microRNAs
	Sponge-like regulatory system decreasing miRNA concentration in the cytoplasm
	Can bind RNA-binding proteins
tRNA	Protein synthesis.
	Post- or pre-transcriptional regulators of gene expression
rRNA	Ribosomal subunits (5S, 16S, 23S, 5.8S, 18S and 28S)
	Protein synthesis

**Table 2:** LncRNAs classification attending to genomic position.

LncRNA Category		Definition	Example
Genic	Genic intronic lncRNA	LncRNA overlapping a protein-coding intron at one or more nucleotides	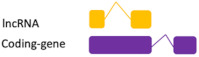
	Genic exonic lncRNA	LncRNA overlapping a protein-coding exon at one or more nucleotides	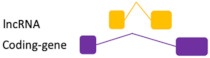
	Nested lncRNA	LncRNA genes contained entirely within protein-coding transcripts	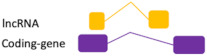
	Overlapping lncRNA	LncRNA genes sharing one or more nucleotides with coding genes	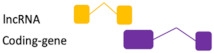
Intergenic	LincRNA	Long-intergenic non-coding RNA. No overlapping, no close to a protein-coding transcript	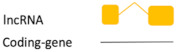
	Same strand	LincRNA within 50 kb of and transcribed from the same strand and in the same direction as the nearest protein-coding transcript	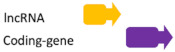
	Convergent	LincRNA within 50 kb of and transcribed head-to-head with the nearest protein-coding transcript	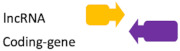
	Divergent	LincRNA within 50 kb of and transcribed tail-to-tail with the nearest protein-coding transcript	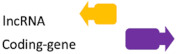

## Supplementary Materials

The following are available online at https://www.mdpi.com/2311-553X/7/1/17/s1, Table S1: Summary table of the ncRNAS cited in the text. The ncRNAs are organised by category (miRNA, lncRNA, piwi or circRNA). The following information is specified: category, chromosomic location, associated process or disease in the literature and related reference number.
