# Supplementary material for: The Implications of ncRNAs in the Development of Human Diseases
Source: Noncoding RNA. 2021 Feb 24;7(1):17. doi: 10.3390/ncrna7010017 (PMC8006041; doi:10.3390/ncrna7010017)
Supplement: Supplementary file 1 [file ncrna-07-00017-s001.pdf]

Supplementary table 1. Summary table of the ncRNAS cited in the text. The ncRNAs are organised by category (miRNA, lncRNA, piwi or circRNA). The following information is specified: category, chromosomic location, associated process or disease in the literature and related reference number.

Gene Symbol	Category	Chomosomic Location	Associated Process or Disease	Reference
Circ-Foxo3	circRNA	6:108984657-108986092	Cell Proliferation	66
CDR1AS/ CIRS-7	circRNA	X:140,781,059-140,783,174	Parkinson, Alzheimer Myocardial infarction	61,119,139
PVT1	lncRNA	8:127794533-128101253	Cancer: breast	93
ANRIL	lncRNA	9:21994778-22032956	Atherosclerosis	130,131
MALAT1	lncRNA	11:65496267-65509085	Atherosclerosis, Myocardial infarction	130,131
KCNQ1OT1	lncRNA	11:2640537-2699998	Atherosclerosis, Myocardial infarction	130,131
aHIF	lncRNA	14:61747039-61749089	Myocardial infarction	130,131
MIAT	lncRNA	22:26646428-26665877	Myocardial infarction	130,131
ZFAS1	lncRNA	20:49278178-49289260	Acute Myocardial infarction	139
UCA1	lncRNA	19:15828947-15835420	Acute Myocardial infarction	140
MEG3	lncRNA	14:100779410-100829185	Cancer: cervical, bladder	90, 91, 92
RMST	lncRNA	12:97431653-97565015	Neuronal differentiation	111
TUNA	lncRNA	14:95876258-95898568	Neuronal differentiation	11
UCHL1-AS	lncRNA	4:41220074-41256727	Parkinson	43,118
ncRNA 17A	ncRNA	9:98542043-98541873	Alzheimer	121, 122
lncRNA-fendrr	lncRNA	16:86487737-86509099	Heart differentiation	128
lncRNA-HBL1	lncRNA	13:54113951-54116706	Ischemic heart failure	129
lncRNA-Bvhrt	lncRNA, mice	18:61799307-61807126	Cardiac development	126, 127
miR-126-3p	miRNA	9:136670653-136670674	Ischemia, myocadial fibrosis, ventricular remodeling, arrythmia, heart failure	132
miR-21-5p	miRNA	17:59841273-5984129	Ischemia, myocadial fibrosis, ventricular remodeling, arrythmia, heart failure	132
let-7g-5p	miRNA	3:52268336-52268357	Myocardial infarction	138
miR-106a-5p	miRNA	X:134170244-134170266	Myocardial infarction	138
miR-424-5p	miRNA	X:134546680-134546701	Myocardial infarction	138
miR-144-3	miRNA	17:28861548-288615	Myocardial infarction	138

p		67		
miR-660-5p	miRNA	X:50013256-50013277	Myocardial infarction	138
miR-192-5p	miRNA	11:64891203-64891223	Heart failure	138
miR-194-5p	miRNA	1:220118206-220118227;11:64891404-64891425	Heart failure	138
miR-34a-5p	miRNA	1:9151735-9151756	Heart failure	138
miR-204-5p	miRNA	9:70810031-70810052	Type 1 Diabetes	142
miR-211-5p	miRNA	15:31065095-31065116	Type 1 Diabetes	142
miR-30d	miRNA	8:134804876-13480494	Diabetes	143
miR-375	miRNA	2:219001645-219001708	Diabetes	144-146
miR-200	miRNA	1:1167863-1167952; 12:6963699-6963766	Cancer: breast, IgA neutropathy	93,149
miR-155	miRNA	21:25573980-25574044	IgA neutropathy	150
miR-146a	miRNA	5:160485352160485450	IgA neutropathy	150
miR-17/92	miRNA	13:91350605-91350688	Cancer	76 ,77
miR-143/145	miRNA	5:-149428918-149429023;5149430646-149430733	Cancer: colon, breast, lung, head and neck, bladder	78
miR-210	miRNA	11:568089-568198	Cancer	135,136
miR-124	miRNA	8:9903388-9903472; 8:64379149-6437925;20:63178500-6317858	Neurogenesis	110
miR-132	miRNA	17:2049908-2050008	Neurogenesis, Synaptic connections	110,112
miR-9	miRNA	1:156420341-156420429;5:88666853-88666939;15:89368017-89368106	Synaptic connections	112
miR-134	miRNA	14:101054687-101054759	Synaptic connections	112
miR-138	miRNA	3:44114212-44114310;16:56858518-56858601	Synaptic connections	112
miR-153	miRNA	2:219294111-21929420;7:157574336-157574422	Parkinson	113, 114
miR-7	miRNA	9:83969748-83969857;5:88666853-88666939;15:89368017-89368106	Parkinson	61,112, 113
miR-106a	miRNA	X:134170198-134170278	Alzheimer	123, 124
miR-520c	miRNA	19:53707453-53707539	Alzheimer	123, 124
miR-20a	miRNA	13:91351065-91351135	Alzheimer	123, 124

miR-17	miRNA	13:91350605-91350688	Alzheimer	123, 124
miR-16	miRNA	13:50048973-50049061;3:160404745-160404825	Alzheimer	123, 124
miR-101	miRNA	1:65058434-65058508;9:4850297-4850375	Alzheimer	123, 124
miR-147	miRNA	9:120244979-120245050;5:hsa-mir-147b	Alzheimer	123, 124
miR-655	miRNA	14:101049550-101049646	Alzheimer	123, 124
miR-323-3p	miRNA	14:101025732-101025817	Alzheimer	123, 124
miR-644	miRNA	20:34466325-34466418	Alzheimer	123, 124
miR-153	miRNA	2:219294111-219294200;7:157574336-157574422	Alzheimer	123, 124
miR-21	miRNA	17:59841266-59841337	Atherosclerosis	135, 136, 180-183
miR-210	miRNA	11:568089-568198	Cancer, Atherosclerosis	82,135 136
miR-29	miRNA, human and mice	7:130876747-130876881;1:207801852-207801939	Diabetes	147
miR-33	miRNA, mice	22:41900949-41900969	Diabetes	148
piR-4987	piRNA	9:113108854-1131088863	Cancer: breast	101
piR-20365	piRNA	1:23413356-232423381	Cancer: breast	101
piR-20485	piRNA	3:126598591-126598620	Cancer: breast	101
piR-20582	piRNA	19:34816976:34817006	Cancer: breast	101
